# The IGF1 Receptor Is Involved in Follicle-Stimulating Hormone Signaling in Porcine Neonatal Sertoli Cells

**DOI:** 10.3390/jcm8050577

**Published:** 2019-04-27

**Authors:** Rossella Cannarella, Iva Arato, Rosita A. Condorelli, Giovanni Luca, Federica Barbagallo, Angela Alamo, Catia Bellucci, Cinzia Lilli, Sandro La Vignera, Riccardo Calafiore, Francesca Mancuso, Aldo E. Calogero

**Affiliations:** 1 Department of Clinical and Experimental Medicine, University of Catania, 95123 Catania, Italy; rosita.condorelli@unict.it (R.A.C.); federica.barbagallo11@gmail.com (F.B.); angela.alamo1986@gmail.com (A.A.); sandrolavignera@unict.it (S.L.V.); acaloger@unict.it (A.E.C.); 2 Department of Experimental Medicine, University of Perugia, 06123 Perugia, Italy; iva.arato@libero.it (I.A.); giovanni.luca@unipg.it (G.L.); catia.bellucci@unipg.it (C.B.); cinzia.lilli@unipg.it (C.L.); francesca.mancuso@unipg.it (F.M.); 3 Department of Medicine, University of Perugia, 06123 Perugia, Italy; riccardo.calafiore@unipg.it

**Keywords:** Follicle-stimulating hormone, Insulin-like growth factor 1, Insulin-like growth factor 1 receptor, Sertoli cells, infertility

## Abstract

Experimental evidence has shown that the IGF1 receptor (IGF1R) is involved in testicular development during embryogenesis. More recently, data gathered from mice granulosa cells and zebrafish spermatogonia suggest that IGF1R has a role in Follicle-stimulating hormone (FSH) signaling. No evidence has been reported on this matter in Sertoli cells (SCs) so far. The aim of the study was to evaluate the role, if any, of the IGF1R in FSH signaling in SCs. The effects of FSH exposure on myosin-phosphatase 1 (MYPT1), ERK 1/2, AKT^308^, AKT^473^, c-Jun N-terminal kinase (JNK) phosphorylation and on anti-Müllerian hormone (AMH), inhibin B and FSH receptor (FSHR) mRNA levels were assessed with and without the IGF1R inhibitor NVP-AEW541 in purified and functional porcine neonatal SCs. Pre-treatment with NVP-AEW541 inhibited the FSH-induced MYPT1 and ERK 1/2 phosphorylation, decreased the FSH-dependent Protein kinase B (AKT)^308^ phosphorylation, but did not affect the FSH-induced AKT^473^ and JNK phosphorylation rate. It also interfered with the FSH-induced AMH and FSHR down-regulation. No influence was observed on the FSH-stimulated Inhibin B gene expression. Conclusion. These findings support the role of theIGF1R in FSH signaling in porcine SCs. The possible influence of IGF1 stimulation on the FSH-mediated effects on SCs should be further explored.

## 1. Introduction

Follicle-stimulating hormone (FSH) is required for normal spermatogenesis [1]. A deeper insight into the molecular mechanisms involved in FSH signaling in Sertoli cells (SCs) might help to elucidate some cases of unexplained male infertility. As for many G protein-coupled receptors (GPCRs), the FSH receptor (FSHR), once over stimulated by FSH, triggers Gαs, which activates the adenylate cyclase, resulting in increased intracellular cAMP levels. The latter leads to protein kinase A (PKA) activation, which in turn stimulates many different transcription factors [2].

A number of studies have assigned a role in SC function to the insulin-like growth factor 1 receptor (IGF1R), which belongs to the tyrosine kinases receptor family [3]. Accordingly, the IGF1R is expressed in SCs and is required for testis development [4] and SC proliferation [5].

The phosphatidylinositol-3 kinase (PI3K) signaling, involving AKT phosphorylation, is required for cell transcription, translation, proliferation and apoptosis [6]. PI3K, which is classically activated by tyrosine kinases receptors such as IGF1R [7], is also stimulated by several GPCRs. The mechanisms through which GPCRs are able to activate PI3K are less understood compared with the classical activation by tyrosine kinases receptors [6]. The PI3K/AKT pathway has been showed to be required for the FSH-dependent gene expression regulation [8]. Recently, FSH has been shown to activate the PI3K in a PKA-dependent manner [9]. Some evidence suggests that the mechanism through which FSH activate the PI3K/AKT signaling may entail the IGF1R. Accordingly, a study carried out in mouse granulosa cells showed a lack of FSH-induced AKT phosphorylation in NVP-AEW541 (an IGF1R inhibitor) pre-treated cells, thus suggesting that the IGF1R is required for FSH signaling [8]. Similar findings have been reported in spermatogonia from zebrafish [10].

The protein phosphatase 1β (PP1β) has been regarded as the possible hub linking between the FSH and the IGF1R signaling in granulosa cells [8]. PP1 is an ubiquitous eukaryotic Ser/Thr phosphatase involved in the regulation of various cell functions. The substrate specificity is given by the binding of the regulatory subunit to the PP1 catalytic subunit (PP1c). The myosin-phosphatase 1 (MYPT1) is a protein made up by three subunits: the PP1c, a targeting/regulatory subunit and a 20kDa subunit of unknown function called M20 [11,12]. PP1 and MYPT1 have been found to be associated with IRS1 in mouse granulosa cells [13]. Furthermore, PKA is known to activate PP1 through MYPT1 phosphorylation [13]. Incubation with tautomycim, a selective PP1β inhibitor, has been shown to inhibit FSH-mediated IRS1 phosphorylation, in the presence of endogenous IGF1 in granulosa cells [8].

The role of the IGF1R in FSH signaling has not been investigated in SCs so far. Therefore, this study was undertaken to explore this topic. To accomplish this, we evaluated the effects of FSH on MYPT1^668^, ERK 1/2, AKT^308^, AKT^473^, JNK phosphorylation in purified and functional porcine neonatal SCs, with and without pre-treatment with the IGF1R inhibitor NVP-AEW541 and the PP1β inhibitor tautomycin. We also investigated whether the FSH-dependent AMH, Inhibin B and FSHR gene expression was influenced by pre-treatment with the IGF1R inhibitor NVP-AEW541. 

## 2. Experimental Section

### 2.1. Ethics Statement

This study was conducted in strict compliance with the Guide for the Care and Use of Laboratory Animals of the National Institutes of Health and Perugia University Animal Care. The protocol was approved by the internal Institutional Ethic Committee (Ministry of Health authorization n. 971/2015-PR, 9/14/2015).

### 2.2. Sertoli Cell Isolation, Culture, Characterization and Function

SCs were obtained from neonatal prepubertal Large White pigs at 7–15 days of age. From each testis, we isolated 60 × 10^6^ SCs with a 95% of purity and a negligible percentage of contaminant cells (Leydig and Peritubular cells < 5%), using established methods [14,15]. Briefly, after removing the fibrous capsule, the testes were finely chopped and digested twice enzymatically, with a mixed solution of trypsin and deoxyribonuclease I (DNase I) in Hank’s Balanced Salt Solution (HBSS; Merck KGaA, Darmstadt, Germany) and collagenase P (Roche Diagnostics S.p.A., Monza, Italy). The tissue pellet was centrifuged passed through a 500-μm pore stainless steel mesh, and then resuspended in glycine to eliminate residual Leydig and peritubular cells [16]. The resulting pellet was collected and maintained in HAM’s F12 medium (Euroclone, Milan, Italy), supplemented with 0.166 nmol l−1 retinoic acid, (Sigma-Aldrich, Darmstadt, Germany) and 5 mL per 500 mL insulin-transferrin-selenium (ITS, Becton Dickinson cat. no. 354352; Franklin Lakes, NJ, USA) in 95% air/5% CO_2_ at 37 °C. After three days in culture, the purity and the functional competence of SC monolayers were performed according to previously established methods [17].

### 2.3. Culture and Treatment

When the SC monolayers were confluent (at three days of culture), they underwent the following treatments: (1) dimethyl sulfoxide (DMSO) or NVP-AE541 for 1 h and then incubated with vehicle or urofollitropin (hpFSH) (Fostimon^®^, IBSA Farmaceutici Srl, Rome, Italy) at the concentration of 50 ng/mL for 15 min; (2) DMSO or 1 µM tautomycin for 5.5 h, followed by vehicle or hpFSH (50 ng/mL) for 15 min; (3) DMSO or 1 µM tautomycin for 5.5 h and NVP-AEW541 for 1 h, followed by vehicle or hpFSH (50 ng/mL) for 15 min, as described elsewhere [16]. Cultures were maintained in humidified atmosphere of 95% air/5% CO_2_ at 34 °C.

### 2.4. Western Blot Analysis

At the end of the incubation period, total cell lysates were collected in radioimmunoprecipitation assay (RIPA) lysis buffer (Santa Cruz Biotechnology Inc., Santa Cruz, CA, USA). The mixture was centrifuged at 1000× *g* (Eppendorf, NY, USA) for 10 min, the supernatant was collected and total protein content was measured by the Bradford method [18]. Sample aliquots were stored at −20 °C for Western blot (WB) analysis. The cell extracts were separated by 4%–12% SDS-PAGE and equal amounts of protein (70 μg protein/lane) were run and blotted on nitrocellulose membranes (BioRad, Hercules, CA, USA). The membranes were incubated overnight in a buffer containing 10 mM Tris(Hydroxymethyl)aminomethane (TRIS), 0.5 M NaCl, 1% (v/v) Tween 20 (Sigma-Aldrich), rabbit 3048 anti-pospho-MYPT1 (Ser 668) (dilution factor 1:1000) (Cell Signaling), rabbit PA5-17164 anti-myosin-phosphatase 1 (MYPT1) (dilution factor 1:1000) (ThermoFisher), rabbit 13038 anti-phospho-AKT (Thr308) (dilution factor 1:1000) (Cell Signaling), rabbit 9271 anti-phospho-AKT (Ser473) (dilution factor 1:1000) (Cell Signaling), rabbit 9272 anti-AKT (dilution factor 1:1000) (Cell Signaling), mouse 05-481 anti-phospho-ERK Kinase1/2 (dilution factor 1:100) (Millipore Merck), ABS44 rabbit anti-ERK 1/2 (dilution factor 1:2000) (Millipore Merck), rabbit 07-175 anti-phospho-JNK (Thr18/Tyr185,Thr221/Tyr223) (dilution factor 1:500) (Millipore Merck), rabbit 06-748 anti-JNK (dilution factor 1:1000) (Millipore Merck), mouse anti-Glyceraldehyde-3-Phosphate Dehydrogenase (GADPH) (6C5): sc-32233 (dilution factor 1:200) (Santa Cruz) primary antibodies. Primary antibody binding was then detected by incubating the membranes for an additional 60 min in a buffer containing horseradish peroxidase conjugated anti-rabbit (Sigma-Aldrich; dilution factor, 1:5000) and/or anti-mouse (Santa Cruz Biotechnology Inc.; dilution factor, 1:5000) IgG secondary antibodies. The bands were detected by enhanced chemiluminescence.

### 2.5. Reverse Transcription Polymerase Chain Reaction Analysis

Total RNA was extracted and quantified by reading the optical density at 260 nm. In particular, 2.5 μg of total RNA was subjected to reverse transcription (RT, Thermo Scientific, Waltham, MA, USA) to a final volume of 20 μL. The qPCR was performed using 50 ng of the cDNA prepared by RT and a SYBR Green Master Mix (Stratagene, Amsterdam, The Netherlands–Agilent Technology). This was performed in an Mx3000P cycler (Stratagene), using FAM for detection and ROX as the reference dye.

The following primers were used for real-time PCR analysis: AMH, forward primers 5′-GCGAACTTAGCGTGGACCTG-3′, revers primers 5′-CTTGGCAGTTGTTGGCTTGATATG-3′; Inhibin B, forward primers 5′-TGGCTGGAGTGACTGGAT-3′, revers primers 5′-CCGTGTGGAAGGATGAGG-3′; FSHR forward primers 5′-TTTCACAGTCGCCCTCTTTCCC-3′, revers primers 5′-TGAGTATAGCAGCCACAGATGACC-3′; actin, forward primers 5′-ATGGTGGGTATGGGTCAGAA-3′, revers primers 5′-CTTCTCCATGTCGTCCCAGT-3′.

### 2.6. Statistical Analysis

Results are shown as mean ± SD throughout the study. Data were analyzed for statistical significance by one-way ANOVA, followed by Tukey post hoc test using SPSS 9.0 for Windows (SPSS Inc., Chicago, IL, USA). A statistically significant difference was accepted when the *p* value was lower than 0.05.

## 3. Results

To elucidate whether the IGF1R and PP1β are involved in FSH signaling, we investigated if the FSH-dependent MYPT1, AKT and JNK phosphorylation was affected by pre-treatment with NPV-AEW541 (an IGF1R inhibitor) and/or tautomycin (a PP1β inhibitor). To further analyze the role of the IGF1R on the FSH-dependent AMH and inhibin B gene expression, we evaluated AMH and inhibin B mRNA levels in the FSH-incubated plates, with and without pre-treatment with NPV-AEW541.

### 3.1. Western Blot Analysis

**Figure 1:**
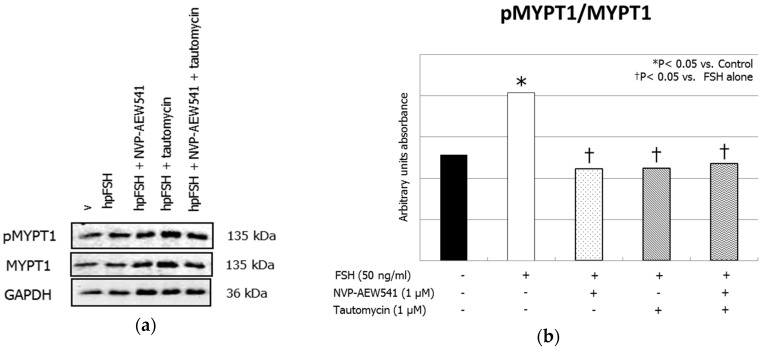
Insulin-like growth factor 1 receptor (IGF1R) is required for the Follicle-stimulating hormone (FSH)-induced myosin-phosphatase 1 (MYPT1) phosphorylation. (**a**) Immunoblots and (**b**) densitometric analysis of phosphorilated myosin-phosphatase 1 (pMYPT1), MYPT1 and Glyceraldehyde-3-Phosphate Dehydrogenase (GADPH) from Sertoli cells alone (control), or incubated with hpFSH alone or pre-treated with the IGF1R inhibitor NVP-AEW541 and/or protein phosphatase 1ß (PP1ß) inhibitor tautomycin and then incubated with hpFSH. Data represent the mean ± standard error of the mean (SEM) (* *p* < 0.05 vs. controls and † *p* < 0.05 vs. FSH treatment alone) (one-way ANOVA) of three independent experiments, each performed in triplicate.

**Figure 2:**
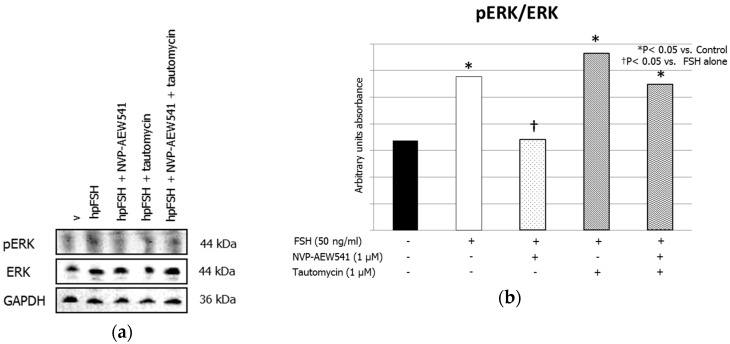
IGF1R is required for the FSH-induced extracellular-signal-regulated kinase (ERK) 1/2 phosphorylation. (**a**) Immunoblots and (**b**) densitometric analysis of the protein bands of pERK1/2, ERK1/2 and Glyceraldehyde-3-Phosphate Dehydrogenase (GADPH) from SCs alone (control) or incubated with hpFSH alone or pre-treated with the IGF1R inhibitor NVP-AEW541 and/or PP1ß inhibitor tautomycin and then incubated with hpFSH. Data represent the mean ± SEM (* *p* < 0.05 vs. controls and † *p* < 0.05 vs. FSH treatment alone) (one-way ANOVA) of three independent experiments, each performed in triplicate.

**Figure 3:**
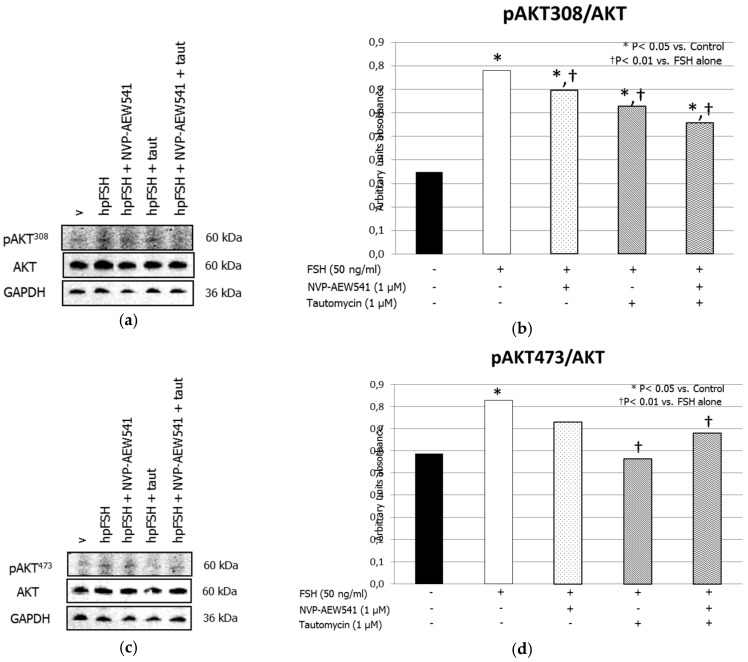
IGF1R is involved in the FSH-induced Protein kinase B (AKT) (Thr308) phosphorylation. (**a**) Immunoblots and (**b**) densitometric analysis of the protein bands of pAKT^308^, AKT and GADPH and (**c**) Immunoblots and (**d**) densitometric analysis of the protein bands of pAKT^473^, AKT and GADPH from SCs alone (control), or incubated with hpFSH alone or pre-treated with the IGF1R inhibitor NVP-AEW541 and/or PP1ß inhibitor tautomycin and then incubated with hpFSH. Data represent the mean ± SEM (* *p* < 0.05 vs. control and † *p* < 0.01 vs. FSH treatment alone) (one-way ANOVA) of three independent experiments, each performed in triplicate.

**Figure 4:**
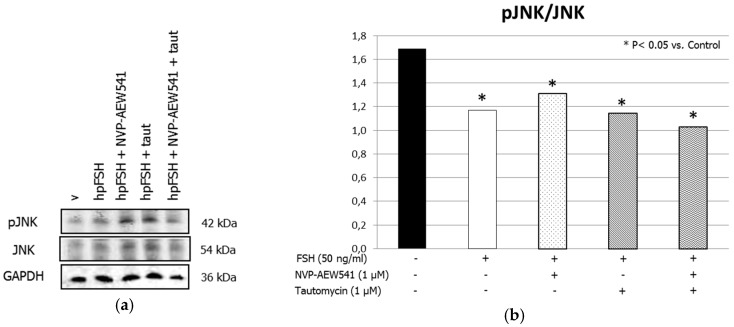
IGF1R does not influence the FSH-induced JNK (Thr18/Tyr185, Thr221/Tyr223) dephosphorylation. (**a**) Immunoblots and (**b**) densitometric analysis of the protein bands of pJNK, JNK and GADPH from SCs alone (control), or incubated with hpFSH alone or pre-treated with the IGF1R inhibitor NVP-AEW541 and/or PP1ß inhibitor tautomycin and then incubated with hpFSH. Data represent the mean ± SEM (* *p* < 0.05 vs. control) (one-way ANOVA) of three independent experiments, each performed in triplicate.

Treatment with FSH increased the MYPT1668/MYPT1 phosphorylation ratio. This effect was inhibited by pre-treatment with NVP-AEW541 and/or tautomycin (Figure 1, panels a and b). FSH increased ERK1/2 phosphorylation. Pre-treatment with NVP-AEW541 resulted in the inhibition of the FSH-induced ERK 1/2 phosphorylation. Tautomycin did not have any effect (Figure 2, panels a and b). Treatment with FSH increased AKT^308^/AKT ratio, but by a lesser extent after pre-treatment with NVP-AEW541 and/or tautomycin (Figure 3, panels a and b). FSH also increased AKT^473^/AKT phosphorylation ratio. Pre-treatment with NVP-AEW541and/or tautomycin hindered the FSH-stimulated AKT^473^ phosphorylation rate (Figure 3, panels c and d). Finally, FSH decreased JNK phosphorylation rate. This effect was not influenced by pre-treatment with NVP-AEW541 and/or tautomycin (Figure 4).

### 3.2. mRNA Analysis

**Figure 5:**
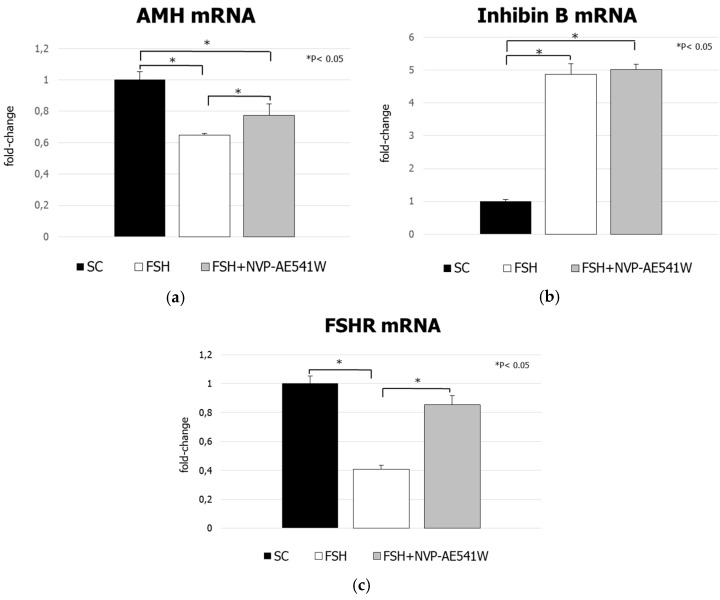
Reverse transcription polymerase chain reaction analysis of (**a**) anti-Müllerian hormone, (**b**) inhibin B and (**c**) FSHR gene expression. Data represent the mean ± SD (* *p* < 0.05 vs. control or FSH treatment) (one-way ANOVA) of three independent experiments, each performed in triplicate.

Treatment with FSH decreased significantly AMH mRNA levels compared to control (−54.7%, *p* < 0.01). The extent of this inhibition was lower in pre-treated cultures (−22.6%, *p* < 0.05 vs. control) (Figure 5, panel a). FSH increased inhibin B mRNA levels compared to control (+487%, *p* < 0.01). These effects were not influenced by pre-treatment with NVP-AEW541 (+501%, *p* < 0.01 vs. control) (Figure 5, panel b). FSH exposure also decreased FSHR mRNA levels compared to control (−59.1%, *p* < 0.01). This was inhibited by NVP-AEW541 pre-treatment (−15%, *p* > 0.05 vs. control) (Figure 5, panel c).

## 4. Discussion

We have recently reviewed the effects of the IGF system (mainly IGF1, IGF2 and IGF1R) on testicular differentiation and function in several species including the human one [3]. Altogether, in-vitro evidence suggests that IGF1 and its receptor play a role in basal and FSH-mediated SC or germ cell proliferation [5,10].

Data from mouse granulosa cells have shown the involvement of the IGF1R in FSH signaling. In greater details, IGF1R was required for the FSH-dependent AKT^308^, AKT^473^, IRS^1989^ and IGF1RTyr^1135/1136^ phosphorylation [8]. In addition, pre-treatment with tautomycin, a PP1 inhibitor, suppressed the FSH-induced AKT^308^, AKT^473^, IRS^1989^ phosphorylation, thus suggesting that the serine/threonine (Ser/Thr) PP1 is necessary for the FSH-mediated IRS1 and AKT phosphorylation. Data from zebrafish confirmed such findings. Indeed, incubation with FSH (promoting type A and B spermatogonia proliferation) increased the IGF3 (a fish-specific member of the IGF family) expression by the PKA and ERK pathways. The FSH-induced proliferation was hindered by the incubation with an IGF3R inhibitor in type A spermatogonia [10].

The results of the present study seem to confirm the existence of an interplay between FSH and IGF1R signaling in SCs. Accordingly, we found that both PP1 and IGF1R inhibition resulted in a lack of FSH-mediated MYPT1 phosphorylation in porcine SCs. Therefore, it may be hypothesized that, similarly to what reported in granulosa cells, IGF1R, IRS1, PP1 and MYPT1 gather together in a molecular complex that requires a conserved tyrosine kinase activity of PP1 and IGF1R to achieve a normal MYPT1 phosphorylation rate under FSH stimulation.

Moreover, in porcine SCs, the FSH-stimulated ERK1/2 phosphorylation occur with an IGF1R-dependent mechanism. PP1 showed to be replaceable for this outcome. Curiously, the double PP1 and IGF1R inhibition did not affect the FSH ability to phosphorylate ERK1/2. In addition, the FSH-dependent AKT phosphorylation was affected by PP1 or IGF1R inhibition. This was expectable since the phosphorylation of AKT reflects the degree of PI3K activation, which in turn is triggered by the IGF1R [19,20].

For the first time, we have also observed that a JNK dephosphorylation occurred after the exposure to FSH. This outcome was not affected by PP1 or IGF1R inhibition. Finally, the FSH-induced downregulation of AMH and FSHR gene expression was IGF1R-mediated. By contrast, IGF1R did not interfere with the FSH-mediated enhancement of inhibin B gene expression.

Porcine SCs have a high degree of similarity with the human ones. Indeed, they have been used in human transplantation experimental protocols for the treatment of patients with type I diabetes mellitus without the need of immunosuppressive therapy [21,22,23]. Given this similarity, the existence of an interplay between the IGF1R and FSH signaling in human SCs cannot be excluded. According the positive correlation between IGF1 levels and testicular volume in men supports this hypothesis [24]. In addition, the testicular to pubic bone distance, which has been proposed as a marker of testicular post-natal function, has been found to positively correlate with IGF1 in children [25]. The understanding of the role of IGF1 and its receptor on human SC physiology, as well as the possible influence on FSH effects, might help to elucidate some cases of unexplained male infertility. Data on infertile women suggest that this topic deserve further investigation. In fact, a meta-analytic study showed the efficacy of GH administration (which in turn increases IGF1 levels) in combination with gonadotropins in poor responder women undergoing to controlled ovarian hyper-stimulation for assisted reproductive technologies compared to standard therapy [26].

Our results need to be taken with care since the present experimental model does not resemble the complexity of the testicular tissue. Indeed, being an in-vitro study carried out only on SCs, we do not know how the paracrine cross-talk with Leydig cells might impact the SCs responsiveness to FSH in the presence of IGF1R inhibition in vivo. Second, we referred to protocols adopted in granulosa cells for doses and time of incubation, but dose-response analysis of tautomycin, NVP-AEW541 and FSH incubation in SCs are warranted. All these limitations should be taken into consideration in further experimental studies.

## 5. Conclusions

In conclusion, the results of this study suggest that IGF1R has a role in the modulation of FSH signaling in porcine SCs. The effects of IGF1 on SC physiology deserve further investigation.
